# Comparison between Ultrasound- and Microwave-Assisted Extraction Methods to Determine Phenolic Compounds in Barley (*Hordeum vulgare* L.)

**DOI:** 10.3390/foods12142638

**Published:** 2023-07-08

**Authors:** María Álvarez-Romero, Ana Ruíz-Rodríguez, Gerardo F. Barbero, Mercedes Vázquez-Espinosa, Fouad El-Mansouri, Jamal Brigui, Miguel Palma

**Affiliations:** 1 Department of Analytical Chemistry, Center of Agri-Food and Wine Research (IVAGRO), Faculty of Science, University of Cadiz, 11510 Puerto Real, Spain; maria.alvarez@uca.es (M.Á.-R.); miguel.palma@uca.es (M.P.); gerardo.fernandez@uca.es (G.F.B.); mercedes.vazquez@uca.es (M.V.-E.); 2 Research Team: Materials, Environment and Sustainable Development (MEDD), Faculty of Sciences and Techniques of Tangier, Abdelmalek Essaâdi University, Tangier BP 416, Morocco; fouad.elmansouri@etu.uae.ac.ma (F.E.-M.); j.brigui@fstt.ac.ma (J.B.)

**Keywords:** barley, phenolic compounds, antioxidant capacity, Box–Behnken design, microwave-assisted extraction, ultrasound-assisted extraction

## Abstract

Barley (*Hordeum vulgare* L.) is one of the major cereal crops worldwide. It is grown not only to be used as fodder but also for human consumption. Barley grains are a great source of phenolic compounds, which are particularly interesting for their health-promoting antioxidant properties, among other benefits. Two extraction methods, namely ultrasound-assisted extraction (UAE) and microwave-assisted extraction (MAE), have been optimized and compared by using Box–Behnken design (BBD) to determine both the antioxidant power and the phenolic compound levels of the extracts. Three variables have been assessed based on these designs: solvent composition (% MeOH in water), temperature (°C), and sample-to-solvent ratio (mg sample mL^−1^ solvent). The solvent composition used and the interaction between the solvent and the temperature were the most significant variables in terms of recovery of phenolic compounds and antioxidant capacity of the extracts. Short extraction times, a high precision level, and good recoveries have been confirmed for both methods. Moreover, they were successfully applied to several samples. Significant differences regarding the level of phenolic compounds and antioxidant power were revealed when analyzing three different barley varieties. Specifically, the amounts of phenolic compounds ranged from 1.08 to 1.81 mg gallic acid equivalent g^−1^ barley, while their antioxidant capacity ranged from 1.35 to 2.06 mg Trolox equivalent g^−1^ barley, depending on the barley variety. Finally, MAE was found to be slightly more efficient than UAE, presenting higher levels of phenolic compounds in the extracts.

## 1. Introduction

Barley is grown and used for both animal and human consumption. For example, it is grown as animal feed because of its high protein content [1]. It is also cultivated for human consumption or as a source of malt for the production of beer or other products such as malt syrup, malted milk, etc. [2]. Its grain is a great source of vitamins, amino acids such as tryptophan, and other substances with interesting antioxidant properties, such as phenolic compounds [3]. Antioxidant substances are those that retard cellular aging by eliminating the free radicals that are generated during oxidation reactions [4,5]. Phenolic compounds are some of the most important antioxidants that can be found in plants. These are organic compounds that contain in their molecular structure an aromatic ring and several phenolic radicals that donate electrons to the free radicals. They exhibit anti-inflammatory, antioxidant, anti-allergenic, and anticarcinogenic properties, among others [6,7,8].

Some of the phenolic compounds found in barley include the following acids: ferulic, caffeic, vanillic, and syringic acid [9,10]; additionally, several flavonoids have been reported, including catechin, epicatechin, kaempferol, quercetin, and rutin [11]. The content and distribution of these phenolic compounds vary depending on the barley variety and growing conditions. Usually, bound phenolics are found in larger levels than free phenolics compounds. The analytical methods to extract either free phenolic compounds or bound phenolic compounds are the same, but with some hydrolysis process applied to free the bound phenolic compounds before the extraction [12]. Several studies have reported that barley phenolic compounds have significant antioxidant power. It has been found that the free phenolic content of barley bran extract was positively correlated with its antioxidant power, as measured by FRAP, ABTS, or DPPH assays [13].

Extraction is the first step for the separation of compounds of interest from any solid matrix. Therefore, rapid, effective, and reliable methods for the extraction of such compounds are required. Conventional extraction methods such as shaking [14] or Soxhlet extraction [15] have been previously applied to this matrix. Moreover, the extraction process from a solid sample can be accelerated and improved using different techniques such as ultrasound-assisted extraction [16], microwave-assisted extraction [17], pulsed field [18], solid–liquid extraction [19], or supercritical fluid extraction [20]. Among the extraction methodologies employed, UAE and MAE are considered to be very good alternatives for the extraction of compounds with high bioactivity effects. They present some advantages such as their simplicity, speed, greater efficiency, and performance and guarantee a high yield and quality of the extract [21].

Ultrasound-assisted extraction (UAE) consists of the application of waves with a frequency between 20,000 and 100,000 Hz to trigger the phenomenon known as “cavitation”, which produces bubbles in the solid–liquid interface. These bubbles collapse with the solvent and break the walls of the sample matrix, which facilitates the release of the analytes [22]. Cavitation provides energy at the contact interface, which results in greater yields in shorter times with smaller amounts of solvents. Due to these advantages, this technique has allowed for the development of several methods enabling the separation of phenolic compounds or antioxidants from their matrix [23,24]. Although previous studies on the phenolic compounds in barley grains have been conducted with the assistance of UAE, the extraction method is still to be optimized [25].

Microwave-assisted extraction (MAE) uses high-frequency waves (0.3 and 300 GHz) to create perpendicularly oscillating electric and magnetic fields [26]. As the intermolecular forces fail to keep in line with these electromagnetic fields because of inertial forces, heat is generated [27]. The samples are therefore heated from their inside to their outside, which favors the penetration of the solvent into the matrix and the release of the analytes [28]. This method is often referred to as a “cold” technique, as the extracted compounds can be quickly transferred into another medium at lower temperature [29]. In recent years, it has been applied to the extraction of antioxidant compounds because of its limited time and solvent demands [30].

This study aims to optimize the extraction methods that allow to determine the levels of free phenolic compounds present in barley and to evaluate their antioxidant power. Bound phenolic compounds were not evaluated using the proposed methods as no hydrolysis procedures were applied. However, the new methods could be applied after a hydrolysis step. In addition, the developed and optimized methods can be subsequently applied to all existing barley varieties and allow extraction of the highest concentration of the free phenolic compounds that are of high interest due to their multiple benefits related to human health.

## 2. Materials and Methods

### 2.1. Reagents

The reagents used for this study of phenolic compounds and antioxidant capacity were Milli-Q water from a Milli-Q water purification system (Millipore, Bedford, MA, USA), methanol (Fischer Chemical, Loughborough, UK), anhydrous sodium carbonate (Panreac Química S.A.U., Castellar del Vallés, Barcelona, Spain), gallic acid, 2,2-diphenyl-1-picrylhydrazyl (DPPH) and 6-hydroxy-2,5,7,8-tetramethylchroman-2-carboxylic acid (Trolox) (Sigma-Aldrich Chemical Co., St. Louis, MO, USA), and Folin–Ciocalteu reagent (Merck KGaA, EMD Millipore Corporation, Darmstadt, Germany).

### 2.2. Sample Preparation

Three commercial types of barley (IPA, Weissbier, and Pale Ale) were obtained from Cervezanía (Seville, Spain). The three types are used for beer production. The samples were mixed and milled using a ZM200 mill (Retsch-Allee 1–5, Haan, Germany) fitted with a 0.25 µm sieve, and the milled mixture was stored at room temperature until analysis.

### 2.3. Ultrasound-Assisted Extraction (UAE)

A Sonopuls Ultrasonic Homogeniser HD4100 (Bandelin, Berlin, Germany) was used for the UAE. The probe was coupled to a thermostatic bath (Frigiterm-10, JP Selecta, Abrera, Spain) to keep a constant temperature according to the experimental conditions. A specific amount of ground sample was weighed for the extraction (according to the design), and 20 mL of the appropriate solvent was added for the extraction process. The amplitude of the equipment was set at 20% of its maximum amplitude (70 W), with an ultrasound cycle of 0.9 s^−1^ for 10 min. Once the extraction had been completed, the extract obtained was centrifuged at 4000 rpm for 5 min. The supernatant was brought up to a volume of 25 mL using Milli-Q water. The extraction procedure was designed according to some previous experience in the extraction of phenolic compounds from different matrices, such as moringa [31] and blackcurrant [32], among others. For the subsequent analysis, the extract was filtered through a nylon syringe filter (0.22 µm).

### 2.4. Microwave-Assisted Extraction (MAE)

The microwave-assisted extractions were carried out using a MARS 6 240/50 1800 W instrument (One Touch Technology, CEM Corporation, Matthews, NC, USA). The corresponding amount of ground sample was weighed directly in the extraction beakers, and 20 mL of the applicable extraction solvent was added according to the experimental conditions. The equipment power was set at 1100 W, and the extraction time was set as 10 min. The operational ranges were set according to the experience of our research group in the extraction of these types of compounds from different matrices such as acai [33], cotton lavender [34], and grape skins [35]. Once the extractions were completed, they were centrifuged at 4000 rpm for 5 min. Similarly to UAE, the supernatant obtained was brought up to a volume of 25 mL using Milli-Q water and filtered through 0.22 µm filters for further analysis.

### 2.5. Analysis of the Phenolic Compounds in the Extracts

The phenolic compound contents in the barley extracts were determined by the Folin–Ciocalteu method [36] since it is widely used in the literature for the determination of such compounds in barley [37,38]. For this purpose, 0.25 mL of sample, previously filtered through a 0.45 µm nylon syringe filter (Nylon Syringe Filter, Filterlab, Barcelona, Spain); 12.5 mL of distilled water; 1.25 mL of Folin–Ciocalteu reagent, and 5 mL of Na_2_CO_3_ solution (20%) were poured into a volumetric flask. Then, an additional amount of distilled water was added to reach a final volume of 25 mL. The mixture was allowed to settle for 30 min, and then, the absorbance was measured at 765 nm using a Cary 60 UV-Vis Spectrophotometer (Agilent Technologies, Santa Clara, CA, USA). The absorbance versus concentration calibration curves produced a regression of y = 0.0529x + 0.0202, with a square linear regression coefficient (R^2^ = 0.9991) and a limit of detection (LOD) of 0.499 mg L^−1^ as well as a limit of quantification (LOQ) of 1.647 mg L^−1^. The results were expressed as mg gallic acid equivalent per gram of barley (mg GAE g^−1^ barley).

### 2.6. Determining the Antioxidant Activity

The DPPH method [39] with some modifications was used to determine the antioxidant activity of the barley extracts. A calibration curve was generated using Trolox as the standard at levels between 0.5 and 100 mg L^−1^. Here, 2 mL of a DPPH solution (6 × 10^−5^ mol L^−1^) and 100 µL of each sample, previously filtered through a 0.45 μm syringe filter (Nylon 15 Syringe Filter, Filterlab, Barcelona, Spain), were used. The sample was allowed to settle for 40 min at room temperature and in the absence of light. Finally, the absorbance was determined at a wavelength of 515 nm. The calibration curve obtained, y = 0.8596x + 0.3090, had a square linear regression coefficient R^2^ = 0.9994, an LOD = 2.096 mg L^−1^, and an LOQ = 6.916 mg L^−1^. The values corresponding to the extract samples were expressed as mg Trolox equivalents per gram of barley (mg TE g^−1^ barley).

### 2.7. Response Surface Methodology (RSM)

A response surface methodology (RSM) based on a Box–Behnken design (BBD) [40] was used for the optimization of the experimental variables involved in the extraction of the phenolic compounds from the barley samples. The independent variables considered for the design were extraction solvent composition (% MeOH in water), extraction temperature, and sample-to-solvent ratio (g sample mL^−1^ solvent). The variables were normalized and coded at 3 levels: +1 (maximum), 0 (medium), and −1 (minimum). In the case of UAEs, the levels to be evaluated regarding each variable were set based on previous works by our research team [31,41] as follows: 20 (−1), 50 (0), and 80 (+1) for the percentage of methanol in water; 0.5:20 (−1), 0.75:20 (0), and 1:20 (+1) for the sample weight/solvent volume ratio (g mL^−1^); and 40 (−1), 55 (0), and 70 (+1) for the temperature (°C). In the same way as for UAE, a BBD was elaborated for MAE. The independent variables evaluated and their ranges were the following: solvent extraction (% MeOH in water): 20 (−1), 50 (0), and 80 (+1); sample-to-solvent ratio (g sample mL^−1^ solvent): 0.5:20 (−1), 0.75:20 (0), and 1:20 (+1); extraction temperature (°C): 50 (−1), 75 (0), and 100 (+1).

### 2.8. Statistical Analysis

The analyses were conducted in triplicate, and the extraction results were expressed as mean ± standard deviation. First, the Shapiro–Wilk test was applied to calculate the normality and homogeneity of their variances. Later, an analysis of variance (ANOVA) was applied, together with Tukey’s test, in order to determine if the different times and the different barley varieties used resulted in statistically significant differences in the levels of phenolic compounds of the extracts at 95% confidence. Thus, the results with a *p*-value < 0.05 were considered statistically different. The software application Statgraphic Centurion V. XVIII (Statgraphics Technologies, Inc., Los Llanos, VA, USA) was used to generate the experimental designs.

## 3. Results

### 3.1. Ultrasound-Assisted Extraction Optimization

#### 3.1.1. Experimental Design

The Box–Behnken design was used due to its high efficiency and the reduced number of experiments required [42]. The effects of three working variables (solvent composition, temperature, and *w*/*v* ratio) on the recovery of phenolic compounds were evaluated. The number of experiments required (N) was determined according to the following equation:

N = 2k(k^−1^) + C_0_ (1)

where k is the number of factors and C_0_ is the number of central points. Three factors and three repetitions of the central point were used, so a design based on 15 experiments was developed.

Once the different experiments were completed and analyzed, it was observed that according to the experimental design, the extraction yields ranged between 0.6950 and 1.0189 mg phenolic compounds per g of barley. The data are shown in Table 1.

Once the ranges were defined, response surface methodology (RSM) and ANOVA were applied to the BBD experimental matrix. The relative error column included in Table 1 displays the relative percentage difference for each of the extraction conditions, i.e., the difference between the actual experimental value and the value calculated by the developed model. It can be seen that it was below 6% in all the cases. This indicates the prediction capabilities of the model. Additionally, the model exhibited 89.86% confidence for the level of phenolic compounds, which means a close agreement between the actual values extracted and those predicted by the model. The standard deviation of the residuals was 0.0549, and the mean absolute error was 0.0266. The *p*-value for the lack of fit (Table 2) was not less than 0.05, so the selected model was considered adequate to describe the observed data. The statistical results obtained by the BBD for the level of phenolic compounds according to each of the effects are shown in Table 2. In this case, three variables, namely weight/volume ratio, temperature, and the quadratic effect of the solvent used, had a *p*-value < 0.05 and were therefore considered significant at the 95% confidence level.

Based on the design, a second-degree polynomial equation was obtained that allowed to model the behavior of the extraction variables and their effect on the recovery of phenolic compounds. This polynomial equation considers only the coefficients of the variables and interactions that have a significant effect on the response to calculate the total phenolic compounds obtained by UAE. Therefore, the non-significant variables and interactions were removed from the resulting model, and this produced similar results to using the full equation. The reduced equation is as follows:

Total phenolic compounds = 0.9880 − 0.0615 × X_2_ + 0.0539 × X_3_ − 0.1150 × X_1_^2^

where X_1_ is the percentage of MeOH in water of the extraction solvent, X_2_ is the weight of the barley used for each extraction per 20 mL of solvent, and X_3_ is the operating temperature in °C.

**Figure 1:**
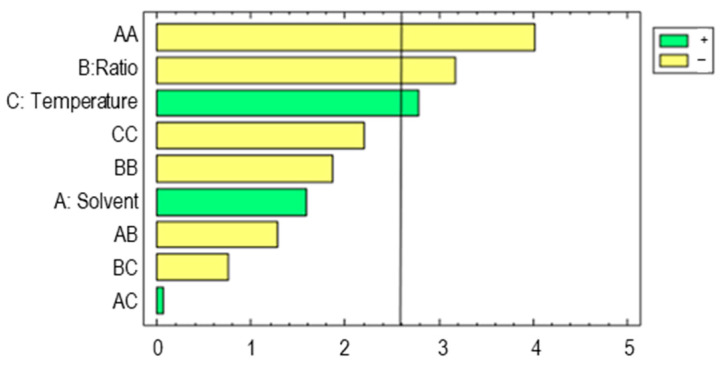
Pareto chart corresponding to the total phenolic compounds extracted from barley by UAE.

These results can be graphically represented. The Pareto chart (Figure 1) shows the significance of each of the variables and their possible interactions in decreasing order. All those variables that exceeded 2.57 have a significant effect on the concentration of phenolic compounds in the extracts.

**Figure 2:**
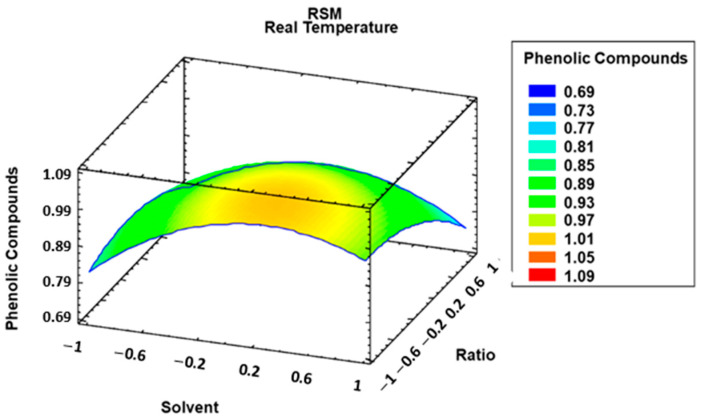
Estimated response surface corresponding to the total phenolic compounds based on the Box–Behnken central composite experimental design considering weight/volume ratio (mg mL^−1^) and solvent composition (% MeOH in water).

The positive signs in Figure 1 (green bars) indicate a direct correlation between the response variable and the working variable, while the negative signs (yellow bars) indicate an inverse correlation. It can, therefore, be seen that the quadratic factor of solvent and *w*/*v* ratio had an inverse effect—i.e., the lower their values were, the more phenolic compounds were extracted. Temperature had a direct correlation with the content of phenolic compounds in the extracts—i.e., the higher the extraction temperature was, the larger the amount of phenolic compounds was. These factors with significant effects were also reported by other studies, such as one on the recovery of antioxidant compounds from black chokeberry [43] where it was revealed that the quadratic product of the solvent had a significant inverse effect, with greater amounts of antioxidant compounds extracted as the quadratic product of the level of methanol in the extraction solvent was reduced. On the other hand, temperature proved to be a significant variable with a direct effect on the extractions obtained, similarly to its effect on the extraction of phenolic compounds from barley. Once the model had been generated, it was used to determine the best values for the extraction variables that produce the largest recovery through a response surface analysis (Figure 2).

The model that was developed allowed to determine the optimum values for the maximum extraction of phenolic compounds. In this case, the following values were set: solvent, 71% MeOH in water; temperature, 64 °C; and weight to liquid ratio, 0.5 g sample in 20 mL of solvent.

Even though the weight/volume ratio employed was the minimum value within the range considered for the experimental design and, as said above [44], this parameter had a significant inverse effect on the response variable, no further tests with lower ratios were performed since they would result in a more challenging quantification of the phenolic compounds [45]. Temperature showed a positive effect. This is because the high temperature levels of the extraction procedures promoted the breaking of van der Waals and dipole–dipole bonds as well as hydrogen bridges. In addition, the low surface tension and viscosity of the solvent enhanced the penetration of the solvent into the matrix, which in turn facilitated quicker releasing of the analytes [46,47]. However, the maximum recovery was not obtained at the highest level for temperature but at 64 °C. It has been also described that temperature can promote the degradation of several phenolics [48].

#### 3.1.2. Determining the Optimum Extraction Time

**Figure 3:**
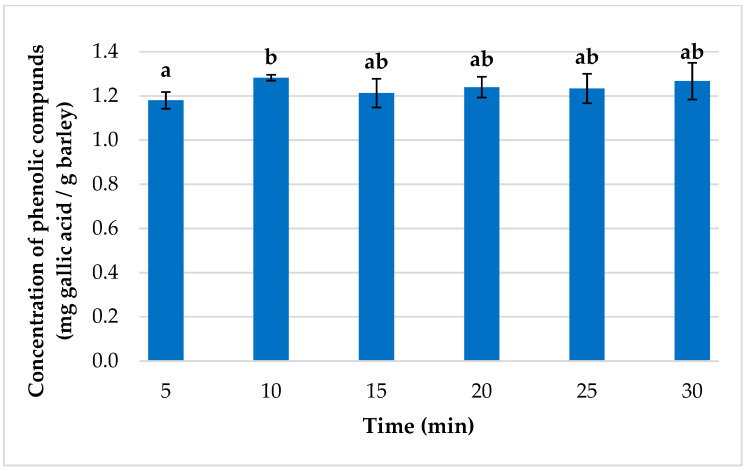
Contents of total phenolic compounds in the extracts obtained using different extraction times (*n* = 3) in the optimized extraction conditions. (A different letter above the bar indicates significant difference between extraction results at 95% confidence level at the extraction time.)

Different extraction times were examined to establish the shortest time that could produce the largest recovery of phenolic compounds in the barley extracts. Times between 5 and 30 min were tested in triplicate (Figure 3). Significant differences were observed between 5 and 10 min, while there were no significant differences between 10 min and any of the longer times used. Therefore, 10 min was established as the optimum extraction time to be used. A reduced extraction time had already been reported as the most appropriate when using this extraction method, which is explained by the large amount of energy involved in the usage of ultrasounds, which, on the one hand, enhances extraction but, on the other, may cause degradation of the phenolic compounds in the matrix [49].

#### 3.1.3. Repeatability, Intermediate Precision, and Recovery

Once the optimal conditions for the extraction method had been established, the repeatability and intermediate precision of the optimized method were determined. In order to determine its repeatability, eight extractions were performed on the same day (*n* = 8), and for the intermediate precision, eight extractions were performed on three consecutive days (*n* = 8 + 8 + 8). The results are shown in Table 3. The results show that the intermediate precision had a coefficient of variation lower than 10%, which is acceptable for an extraction method to be determined by UV-Vis spectroscopy [50].

Finally, a recovery study was conducted on the extraction process. For this purpose, extractions were performed in triplicate under the established optimum extraction conditions, adding 1 mL of a 100 mg L^−1^ gallic acid solution to the sample to be extracted. An average recovery level of 103.48% was registered.

### 3.2. Microwave-Assisted Extraction Optimization

#### 3.2.1. Experimental Design

As with ultrasound, the MAE process was also optimized by means of a Box–Behnken design. The effects of three variables (solvent, temperature, and sample-to-solvent ratio) were evaluated, and a single response—the amount of total phenolic compounds—was monitored. The ranges used for the independent variables considered were 20 (−1), 50 (0), and 80 (+1) for the % MeOH in water; 0.5:20 (−1), 0.75:20 (0), and 1:20 (+1) for the sample-to-solvent ratio (g mL^−1^); and 50 (−1), 75 (0), and 100 (+1) for the temperature (°C). Higher temperatures could be tested for MAE than for UAE since the extraction was carried out in sealed containers. For this study, three factors and three replicates of the central point were used, such that a 15-point experiment was designed.

Once the 15 experiments had been completed, the contents of phenolic compounds in the extracts were determined using the Folin–Ciocalteu method. The results from the experimental design show that the extraction yields were between 0.4662 and 1.5206 mg GAE g^−1^ barley (Table 4), with the maximum amount being greater than that obtained by UAE. Although both techniques actually have a different extraction procedure and mechanism, the stability of the compounds of interest was guaranteed in both methods since the same equipment and similar conditions to a previous study carried out by our research group on the stability of phenolic compounds using UAE and MAE were used [51,52]. In this way, it could be determined which technique is able to extract a higher concentration of these compounds under similar extraction conditions. It can be seen that the difference between the results of the model and the actual experimental data remained acceptably below 10% in most cases. Nevertheless, some differences greater than 10% were registered, particularly in those extraction conditions that resulted in lower recoveries (e.g., experiences 1 and 8). These relative errors were higher than those previously registered for UAE. This mismatch between the results calculated by the model and the actual experimental results can be considered acceptable for this type of experiments, since the relative errors will always be greater in those results corresponding to low recoveries.

When the RSM and ANOVA were performed, the results of which are shown in Table 5, it could be observed that there are four variables (solvent composition, ratio, temperature, and solvent composition–temperature interaction) that have effects with a *p*-value below 0.05, which means that they are significant variables at 95.0% confidence. The analyses indicated that the model had a fit of 93.35% regarding the recovery of free phenolic compounds. The standard deviation of the residuals was 0.1138, and the mean absolute error was 0.0564. In this case, the *p*-value for lack of fit according to the ANOVA was not less than 0.05. Therefore, the model should be considered as suitable to describe the actual data registered.

Similarly to the UAEs, a polynomial equation based on the significant coefficients of the three variables and their interactions should allow to calculate the total phenolic compounds obtained by the MAEs as follows:

Total phenolic compounds = 0.8720 + 0.1629 × X_1_ − 0.1157 × X_2_ + 0.1300 × X_3_ + 0.2635 × X_1_ × X_3_

where X_1_ is the percentage of methanol in the water used as the extraction solvent, X_2_ is the weight of the barley added per 20 mL of solvent, and X_3_ is the extraction temperature in °C.

**Figure 4:**
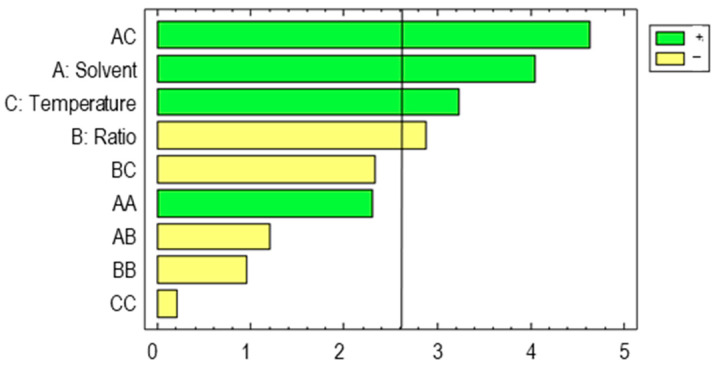
Pareto chart corresponding to the total phenolic compounds in barley MAEs.

It can be seen from the Pareto chart (Figure 4) that the interaction between solvent and temperature (solvent–temperature) as well as the solvent and the temperature factors had a significant positive effect on the extraction of total phenolic compounds in both cases—i.e., the higher the amount of methanol was in the solvent and the higher the temperature was, the greater the level of phenolic compounds in the extracts was.

These results agree with those found in the literature, which suggests that the extraction solvent percentage is one of the variables that most affects the extraction of phenolic compounds [41]. However, in this case, the quadratic effect is contrary to the effect observed in UAEs. In this regard, it should be considered that in UAEs, the solvent is the ultrasound transmission medium; therefore, some opposite effects can appear. Specifically, the level of methanol affects both the solubility of the compounds into the solvent and the transmission of the ultrasounds—i.e., a lower percentage of methanol may facilitate the transmission of the waves but present a lower capacity to solubilize the phenolic compounds [53]. On the other hand, in MAE, since the solvent does not play the same role as in UAE, the composition of the solvent, and therefore its ability to solubilize the compounds to be extracted, becomes the main factor to be considered [54].

**Figure 5:**
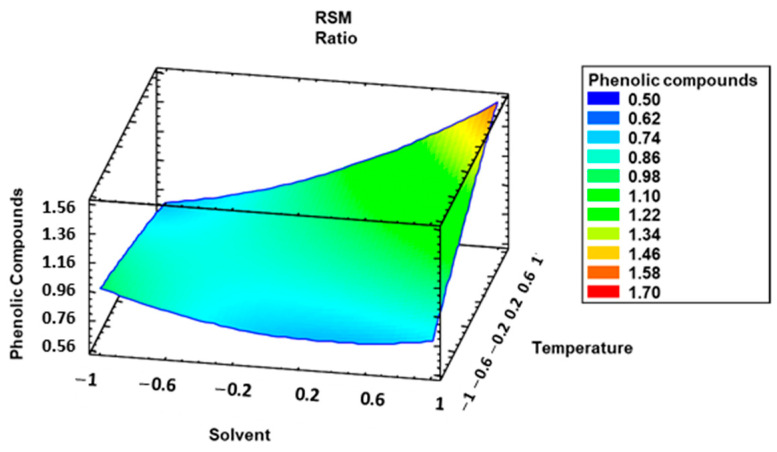
Estimated response surface corresponding to the total phenolic compounds based on the Box–Behnken central composite experimental design considering temperature (°C) and solvent composition (% MeOH in water).

The results obtained from the developed model according to solvent composition and temperature are shown in Figure 5.

In addition, the model developed allowed to determine the optimum values for the maximum extraction of total phenolic compounds. Hence, the following values were established: 80% MeOH extraction solvent, extraction temperature of 100 °C, and 0.5 g sample in 20 mL solvent.

It must be noted that the largest amounts of phenolic compounds were obtained at the maximum values of the range tested in both cases of temperature and solvent percentage, while the weight/volume ratio used was at its minimum value within the range studied. In this regard, the *w*/*v* ratio presented a similar behavior as in the UAEs; hence, a lower amount of barley in the same volume of solvent produced a higher gradient, and that allowed for a larger recovery as it was favored by an enhanced mass transfer. Nevertheless, smaller amounts of sample might result in extracts with a phenolic compound content below the limit of quantification [45]. With regard to temperature, no further studies were performed since it has already been demonstrated that high temperature levels may compromise the stability of the glycosidic phenolic compounds, which might be affected by hydrolysis reactions, while the thermosensitive compounds in the extract would be naturally affected by temperatures in excess of 100 °C [51,55].

Similar values for the optimization variables were obtained by other studies on the extraction of phenolic compounds by MAE from other matrices, such as maqui berry [56]. In that study, the solvent and temperature factors were positively significant, and the weight/volume ratio presented a negative correlation, even though it did not show a significant influence for the confidence level that had been established. In our case, the interaction between solvent and temperature had an inverse relationship, but at no significant level.

**Figure 6:**
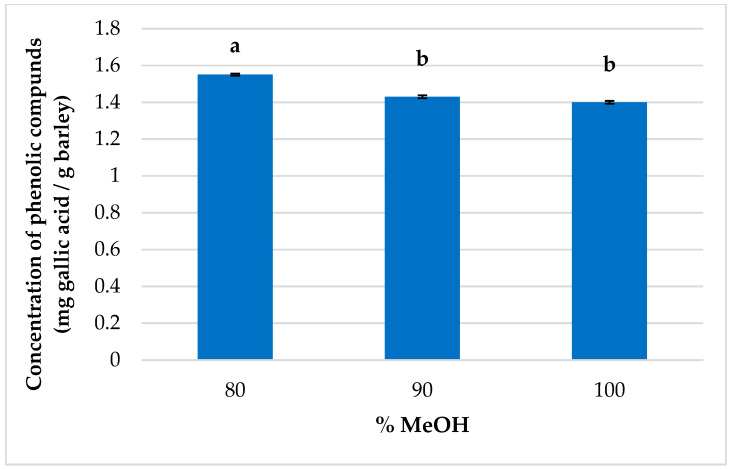
Recovery of gallic acid per gram of barley (*n* = 3) depending on the composition of the solvent used for the MAE (80, 90, and 100% MeOH in water). (A different letter above the bar indicates significant difference between extraction results at 95% confidence level.)

Finally, the optimum composition percentage of the solvent proved to be the maximum value tested for the model. Since higher levels were still applicable, we proceeded to their evaluation. Therefore, triplicate extractions were conducted by applying the best conditions established for the rest of the variables, while only the percentage of methanol in the extraction solvent was modified (80, 90, and 100%). It can be seen from Figure 6 that as the percentage of methanol was increased, a lower recovery of phenolic compounds was achieved.

Various studies have reported that hydroalcoholic solvents with 60–80% MeOH content in water are more efficient than those with 100% MeOH for the extraction of phenolic compounds [57,58]. This is explained by the similar polarity of the compounds and the solvent, but also by the solvent’s ability to penetrate the matrix [59]. Thus, although the similar polarity of methanol and the phenolic compounds facilitates their extraction, water also contributes to facilitate the extraction as it has a greater ability to moisten the solid sample [60].

#### 3.2.2. Optimum Extraction Time

**Figure 7:**
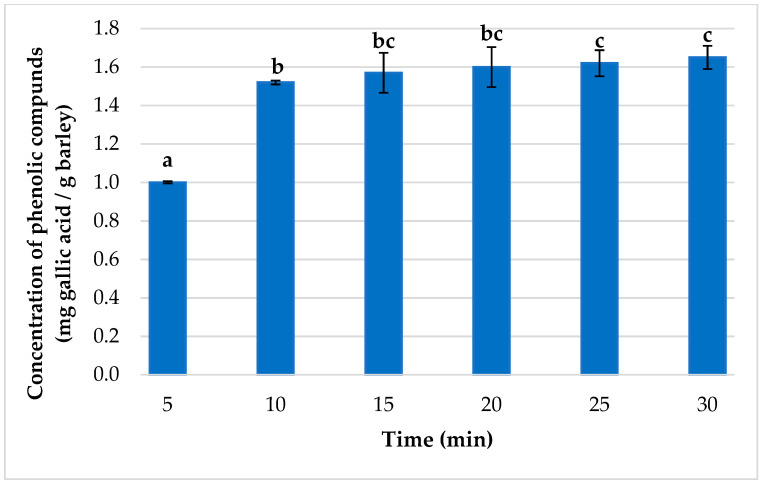
Concentration of total phenolic compounds depending on the extraction times used for MAE (*n* = 3). (A different letter above the bar indicates significant difference between extraction results at 95% confidence.)

To determine the optimal extraction time, triplicate extractions were performed in the range from 5 to 30 min (Figure 7). A growing amount of total phenolic compounds obtained could be observed with longer extraction times. According to Student’s *t*-test (two-tailed), there were no significant differences between 15 and 30 min. Therefore, 15 min was established as the optimum time to extract the maximum amount of phenolic compounds from barley. Thus, the optimal MAE time was longer than that of UAE. Despite the longer time required by MAE, this technique was more efficient for the extraction of phenolic compounds from barley compared to UAE. Similar results have been described in the literature, usually associated with samples whose water content is particularly low [61,62]. It should be noted, in any case, that several MAEs can be carried out simultaneously, so the actual time per sample used would be much shorter, being even shorter than those used for UAEs.

#### 3.2.3. Repeatability, Intermediate Precision, and Recovery

This study was conducted in a similar way to the one performed on UAEs. Table 6 shows that both coefficients were below 5%. The method was therefore considered as precise and repeatable.

In the same way as for the ultrasound-assisted extractions, microwave-assisted extractions were performed in triplicate, adding 1 mL of a 100 mg L^−1^ gallic acid solution to the sample to be extracted. A recovery value of 104.43% was found.

### 3.3. Implementing the Methods with Real Samples

Once the two methods for the extraction of phenolic compounds from barley had been optimized, they were applied to different barley varieties to determine the level of phenolic compounds obtained by both methods. Therefore, 18 extractions were performed (3 real samples, 3 replicates, and 2 methods). The results are shown in Table 7. All the analyses were conducted in triplicate.

First of all, it can be seen that different results were obtained by each method, with significant differences observed between the three barley varieties. The gains were between 25% and 32% when MAE was used to extract the phenolic compounds. It was therefore confirmed that MAE recovered more phenolic compounds than UAE did from the different barley types tested, even if the time required was longer (15 min MAE vs. 10 min UAE). Furthermore, the MAE equipment used in our study would allow up to 40 simultaneous extractions, while the UAE probe was only suitable for single extractions. MAE was therefore confirmed as a much more efficient method to determine the amount of total phenolic compounds in barley, particularly when a large number of samples are to be analyzed.

In view of the data in Table 7, it could be observed that the Pale Ale barley variety produced extracts with a greater level of phenolic compounds, followed by the IPA variety.

### 3.4. Antioxidant Activity

Once the extraction methods had been developed and optimized, the antioxidant capacity of the extracts obtained was determined according to the DPPH method. The results obtained are shown in Table 8. All the determinations were run in triplicate.

It can be observed from Table 8 that, similarly to what occurred with total phenolic compounds, MAEs were capable of extracting a greater amount of antioxidant compounds than UAEs. This applied to the three barley varieties studied. Therefore, just as with the total phenolic compounds, MAE, rather than UAE, would be a more suitable method to evaluate the antioxidant capacity of barley extracts.

It could also be observed that the IPA barley had a larger antioxidant capacity than the other two varieties. Generally speaking, there is some correlation between phenolic compounds content and antioxidant capacity, although this is not strictly proportional, which suggests that there are compounds in the extracts, other than just the phenolic ones, that also have antioxidant power. This fact had already been reported in the literature, as other compounds with antioxidant capacity, such as tryptophan, have been detected in barley [63,64]. This would explain why the antioxidant capacities of the extracts do not fully correlate with their total phenolic compound contents [64].

## 4. Conclusions

Two methods for the extraction of total phenolic compounds from barley, namely UAE and MAE, have been optimized by means of a Box–Behnken response surface design. The percentage of methanol used in the solvent proved to be the most influential variable regarding the amount of phenolic compounds extracted from barley by UAE. MAE, on the other hand, was highly influenced not only by the percentage of methanol in the solvent but also by the extraction temperature. Not only were the recoveries obtained through MAE slightly greater than those achieved through UAE, but this method also allows to conduct a considerable number of simultaneous extractions. Both of them had a high precision, and their suitability has been demonstrated by the successful extraction of phenolic compounds in different barley varieties.

Although it is generally assumed that there is a correlation between the amount of phenolic compounds in extracts and their antioxidant capacity, this is not strictly proportional, which indicates that other antioxidant compounds, and not only phenolic compounds, are present in barley extracts.

It can be concluded from all the results that both methods of UAE and MAE can be used for the determination of free phenolics in barley. Both methods are excellent and affordable alternatives for industry since they can be used for the selection of barley varieties with larger levels of phenolic compounds, which implies better nutritional properties for the health of consumers.

## Figures and Tables

**Table 1:** Working conditions in the experimental design, responses, and relative error.

				Responses (Y_TPC_ (mg g^−1^))		
Experience No.	Factors			Experimental	Predicted	Relative Error * (%)
1	0	−1	−1	0.901	0.859	4.96
2	−1	−1	0	0.805	0.816	1.25
3	−1	1	0	0.806	0.762	5.73
4	−1	0	−1	0.695	0.727	4.46
5	0	0	0	0.930	0.988	5.87
6	1	1	0	0.764	0.754	1.35
7	0	−1	1	1.019	1.008	1.11
8	1	−1	0	0.903	0.947	4.61
9	0	1	−1	0.766	0.777	1.44
10	1	0	1	0.929	0.897	3.62
11	1	0	−1	0.786	0.785	0.14
12	0	1	1	0.801	0.843	5.05
13	−1	0	1	0.830	0.832	0.13
14	0	0	0	1.019	0.988	3.13
15	0	0	0	1.015	0.988	2.74

* Relative error: percentage difference between the absolute actual value of the experiment and the value predicted by the model.

**Table 2:** Estimated regression coefficients and *p*-values of the independent variables in UAEs for the level of phenolic compounds.

Source	Coefficient	Sum of Squares	df	Mean Square	*F*-Value	*p*-Value
Model	0.988					
Solvent	0.031	0.008	1.000	0.008	2.500	0.175
W/v ratio	–0.062	0.030	1.000	0.030	10.060	0.025
Temperature	0.054	0.023	1.000	0.023	7.700	0.039
Solvent × Solvent	–0.115	0.049	1.000	0.049	16.200	0.010
Solvent × *w*/*v* ratio	–0.035	0.005	1.000	0.005	1.620	0.259
Solvent × Temperature	0.002	0.000	1.000	0.000	0.000	0.949
W/v ratio × *w*/*v* ratio	–0.053	0.011	1.000	0.011	3.500	0.120
W/v Ratio × Temperature	–0.021	0.002	1.000	0.002	0.580	0.482
Temperature × Temperature	–0.063	0.015	1.000	0.015	4.840	0.279
Lack of fit		0.010	3.000	0.003	1.320	0.508

**Table 3:** Repeatability and intermediate precision of the optimized method.

	Repeatability	Intermediate Precision
Mean (mg TPC g^−1^ sample)	1.27 ± 0.04	1.26 ± 0.09
CV (%)	3.48	6.78

**Table 4:** Experimental design, experimental and predicted responses, and relative error.

	Factors			Responses (Y_TPC_ (mg g^−1^))		
Experience No.	*X*_1_	*X*_2_	*X*_3_	Experimental	Predicted	Relative Error * (%)
1	0	−1	−1	0.560	0.656	14.6
2	1	1	0	0.865	0.930	6.9
3	0	0	0	0.924	0.872	5.9
4	1	0	−1	0.759	0.765	0.7
5	0	1	−1	0.760	0.690	10.2
6	0	0	0	0.818	0.872	6.2
7	0	1	1	0.780	0.684	14.0
8	−1	1	0	0.640	0.741	13.7
9	1	0	1	1.521	1.552	2.0
10	−1	0	−1	0.997	0.966	3.2
11	1	−1	0	1.400	1.299	7.8
12	−1	0	1	0.705	0.699	0.8
13	0	0	0	0.874	0.872	0.2
14	−1	−1	0	0.900	0.836	7.7
15	0	1	1	1.111	1.181	5.9

* Relative error: percentage difference between the absolute actual values of the experiment and the value predicted by the model.

**Table 5:** Estimated regression coefficients and *p*-values of the independent variables in MAEs of total phenolic compounds.

Source	Coefficient	Sum of Squares	df	Mean Square	*F*-Value	*p*-Value
Model	0.872					
Solvent	0.163	0.212	1.000	0.212	16.400	0.010
Weight/volume ratio	−0.116	0.107	1.000	0.107	8.280	0.035
Temperature	0.130	0.135	1.000	0.135	10.440	0.023
Solvent × Solvent	0.136	0.068	1.000	0.068	5.280	0.070
Solvent × *w*/*v* ratio	−0.069	0.019	1.000	0.019	1.460	0.281
Solvent × Temperature	0.264	0.278	1.000	0.278	21.460	0.006
W/v ratio × *w*/*v* ratio	−0.057	0.012	1.000	0.012	0.920	0.382
W/v ratio × Temperature	−0.133	0.071	1.000	0.071	5.450	0.067
Temperature × Temperature	−0.013	0.001	1.000	0.001	0.040	0.841
Lack-of-fit		0.059	3.000	0.020	7.040	0.127

**Table 6:** Repeatability (*n* = 8) and intermediate precision (*n* = 8 + 8 + 8) of MAEs using the established optimized conditions.

	Repeatability	Intermediate Precision
Mean (mg TPC g^−1^ sample)	1.61 ± 0.07	1.62 ± 0.08
CV (%)	4.28	5.17

**Table 7:** Comparison of the level of phenolic compounds extracted (mg TPC g^−1^ sample) by UAE and MAE from different barley varieties (*n* = 3).

	Weissbier *	IPA *	Pale Ale *
UAE	1.080 ± 0.04	1.213 ± 0.07	1.372 ± 0.06
MAE	1.361 ± 0.08	1.520 ± 0.03	1.811 ± 0.05

* Significant difference between the extraction methods for the same barley variety at 95% confidence.

**Table 8:** Comparison of the antioxidant capacity (mg Trolox equivalent g^−1^ sample) of the extracts obtained through the two extraction methods from the three barley varieties (*n* = 9).

	Weissbier *	IPA *	Pale Ale *
UAEs	1.42 ± 0.14	3.04 ± 0.16	2.73 ± 0.17
MAEs	2.06 ± 0.09	3.37 ± 0.12	3.16 ± 0.15

* Significant differences at 95% confidence between the results obtained by the two extraction methods from the same barley variety.

## Data Availability

All data are included in the manuscript.
